# Room‐Temperature On‐Spin‐Switching and Tuning in a Porphyrin‐Based Multifunctional Interface

**DOI:** 10.1002/smll.202104779

**Published:** 2021-10-12

**Authors:** Henning Maximilian Sturmeit, Iulia Cojocariu, Andreas Windischbacher, Peter Puschnig, Cinthia Piamonteze, Matteo Jugovac, Alessandro Sala, Cristina Africh, Giovanni Comelli, Albano Cossaro, Alberto Verdini, Luca Floreano, Matus Stredansky, Erik Vesselli, Chantal Hohner, Miroslav Kettner, Jörg Libuda, Claus Michael Schneider, Giovanni Zamborlini, Mirko Cinchetti, Vitaliy Feyer

**Affiliations:** ^1^ TU Dortmund University Experimental Physics VI 44227 Dortmund Germany; ^2^ Peter Grünberg Institute (PGI‐6) Jülich Research Centre 52425 Jülich Germany; ^3^ Institute of Physics, University of Graz Karl‐Franzens‐Universität Graz Graz 8010 Austria; ^4^ Swiss Light Source Paul Scherrer Institute Villigen CH‐5232 Switzerland; ^5^ CNR‐IOM TASC Laboratory Trieste 34149 Italy; ^6^ Department of Physics University of Trieste Trieste 34127 Italy; ^7^ Department of Chemical and Pharmaceutical Sciences University of Trieste Trieste 34127 Italy; ^8^ Interface Research and Catalysis Erlangen Center for Interface Research and Catalysis Friedrich‐Alexander University Erlangen‐Nuremberg 91058 Erlangen Germany; ^9^ Faculty of Physics and Center for Nanointegration Duisburg‐Essen (CENIDE) University of Duisburg‐Essen 47048 Duisburg Germany

**Keywords:** charge transfer, functionalization, molecular interfaces, spin switching, spintronics

## Abstract

Molecular interfaces formed between metals and molecular compounds offer a great potential as building blocks for future opto‐electronics and spintronics devices. Here, a combined theoretical and experimental spectro‐microscopy approach is used to show that the charge transfer occurring at the interface between nickel tetraphenyl porphyrins and copper changes both spin and oxidation states of the Ni ion from [Ni(II), *S* **=** 0] to [Ni(I), *S* **=** 1/2]. The chemically active Ni(I), even in a buried multilayer system, can be functionalized with nitrogen dioxide, allowing a selective tuning of the electronic properties of the Ni center that is switched to a [Ni(II), *S* = 1] state. While Ni acts as a reversible spin switch, it is found that the electronic structure of the macrocycle backbone, where the frontier orbitals are mainly localized, remains unaffected. These findings pave the way for using the present porphyrin‐based system as a platform for the realization of multifunctional devices where the magnetism and the optical/transport properties can be controlled simultaneously by independent stimuli.

## Graphical abstract

In multifunctional devices, the magnetic and the optical/transport properties can be controlled simultaneously by independent stimuli. The interface between nickel tetraphenyl porphyrins and copper can be used as a platform for realizing such devices: while nickel acts as a reversible spin switch, the electronic structure of the macrocycle backbone, where the frontier orbitals are localized, remains unaffected.

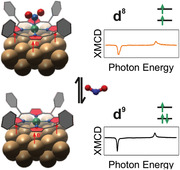

## 1 Introduction

In the last two decades, considerable efforts have been made to stabilize and control the spin and oxidation states in metal ions at the nanoscale in order to design devices with novel functionalities and improved performance. This offers prospects for enhancing the reactivity of single‐atom catalysts,^[^ ^1^ ^]^ developing new strategies for efficient gas separation in metal–organic frameworks,^[^ ^2^ ^]^ building single‐atom magnets capable of storing information,^[^ ^3^ ^]^ as well as realizing spin‐based logic operations.^[^ ^4^ ^]^

One way to stabilize metal ions and to prevent their local magnetic moment from decreasing or being quenched by the interaction with the substrate,^[^ ^5^ ^]^ is to cage them into a ligand field through its lateral unsaturated coordination bonds. This can be achieved using molecular ligands, for example, cyano‐ or carboxylic‐molecular terminations, as demonstrated for Fe, Ni, and Mn ions forming 2D high‐spin metal–organic networks.^[^ ^6^, ^7^, ^8^, ^9^, ^10^ ^]^ An alternative route is to exploit aromatic molecules, such as porphyrins or phthalocyanines, where the metal ion is coordinated in their tetrapyrrolic macrocycle^[^ ^11^, ^12^ ^]^ in different spin configuration states. Long‐range magnetic order can be induced by exploiting the molecule–substrate interaction. In this way, for example, the ferromagnetic coupling of an iron porphyrin with the ferromagnetic film underneath can be established already at room temperature (RT) via superexchange interaction.^[^ ^13^ ^]^

Regarding the control of magnetism at such molecular interfaces, well‐established methods, based on the anchoring of external axial ligands to the ion center,^[^ ^14^ ^]^ allow off‐switching and spin‐tuning transitions.^[^ ^15^, ^16^ ^]^ In contrast, approaches for the realization of a spin‐off → spin‐on transition are limited to the adsorption of molecules at low temperature, since chemical bonding has to overcome the spin‐pairing energy.^[^ ^17^ ^]^ Reversible spin‐switching porphyrin complexes can be obtained by the mechanical movement of an axial ligand strapped to the porphyrin ring.^[^ ^18^ ^]^ This method, however, requires a local voltage pulse generated by a scanning tunneling microscopy (STM) tip. Chemical doping using alkali metals^[^ ^19^ ^]^ is an alternative strategy for manipulating the magnetic moment of a metal ion in tetrapyrrolic compounds. However, this process takes place only at low temperatures and requires a top–down approach to control the alkali atom site.^[^ ^20^ ^]^ In fact, the alkali metal must be precisely placed atop the central ion, often inducing disorder in the molecular array, and therefore a reversibility is hardly achievable.^[^ ^21^ ^]^ Moreover, both functionalization^[^ ^22^ ^]^ and chemical doping^[^ ^20^ ^]^ lead to a decoupling of the molecule from the surface. This, as a result, changes the energy alignment of the molecular orbitals and ultimately affects the carrier injection properties across the molecular interface.^[^ ^23^ ^]^

**Figure 1:**
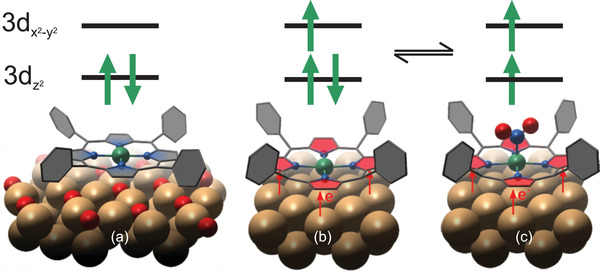
Schematic representation of the behavior of the NiTPP/Cu(100) interface. The magnetic properties are dominated by the central Ni ion (green), while the frontier orbitals that determine the transport, as well as the optical properties, are localized on the macrocycle moiety (depicted in red). In a free NiTPP molecule, the Ni ion is in the [Ni(II), *S* = 0] state. a) The situation is well reproduced when NiTPP is deposited on an oxygen‐reconstructed Cu(100) surface (oxygen atoms in red). b) If NiTPP is in direct contact with the Cu(100) surface, interface charge transfer leads to a reduction of the Ni ion to a [Ni(I), *S* = 1] state. c) Exposure to NO_2_ induces a transition to a new [Ni(II), *S* = 1] state. Although the spin state of NiTPP changes between (b) and (c), the frontier orbitals remain unchanged. They are stabilized by the strong interface charge transfer into the macrocycle moiety.

Here, we introduce a molecular interface that behaves as a reversible spin switch already at RT, whose spin moment is energetically and spatially delocalized from the frontier orbitals, which in turn determine the optical as well as transport properties across the interface. In particular, we show that the charge transfer occurring upon deposition of nickel tetraphenyl porphyrins (NiTPP) on copper(100) reduces the Ni central ion in NiTPP (Ni(II) → Ni(I)) and, at the same time, changes its spin state (*S* = 0 → *S* = 1/2). By means of X‐ray photoelectron spectroscopy (XPS), STM, and infrared reflection absorption spectroscopy (IRAS), we demonstrate that while the Ni(II) is inert, the Ni(I) center becomes highly reactive and can be functionalized with nitrogen dioxide (NO_2_) and this chemical response is preserved even in the buried interface. NO_2_ ligation induces a reversible transition to a new Ni(II), *S* = 1 state. Density functional theory (DFT) calculations, together with X‐ray adsorption measurements at the N K‐edge, show that, while the Ni acts as a switchable spin center, the energy level alignment of the frontier orbitals, which are mainly localized at the macrocycle moiety, remains unaltered during the spin switching. Such intriguing behavior, that paves the way for the utilization of the NiTPP/Cu interface in future multifunctional molecular devices, is schematized in **Figure** **1**.

When a single layer of NiTPP molecules is deposited on a copper (100) surface, the strong interaction at the interface leads to a massive charge transfer, with twofold consequences: i) the reduction of the central Ni(II) ion of the free NiTPP molecule, that stabilizes the uncommon +1 oxidation state Ni(I);^[^ ^24^ ^]^ ii) the filling of the lowest unoccupied molecular orbitals (LUMOs) up to the LUMO+3.^[^ ^25^ ^]^

## 2 Results

### 2.1 Reactivity of the Ni(I) Ion

At first, we demonstrate the extreme reactivity of the Ni(I) ion, by comparing it to the chemically inert Ni(II). To do so, we exposed the NiTPP/Cu(100) interface to increasing doses of NO_2_, which is a strongly oxidizing gaseous agent. As a counter experiment, we selected an interface where the porphyrin molecule is fully decoupled from the substrate underneath, and the +2 state of the Ni ion (Ni(II)) is preserved (similar to the multilayer case^[^ ^24^ ^]^). This can be achieved by depositing the NiTPP on a (√2 × 2√2)R45°‐oxygen‐reconstructed copper(100) surface (for simplicity referred to as O–Cu(100) in the following), where the covalent nature of the Cu–O interaction yields a strong localization of the surface electrons, inhibiting the charge transfer from the substrate to the molecular film.^[^ ^26^, ^27^ ^]^ Further details on the preparation of the different interfaces can be found in the Experimental Section.

**Figure 2:**
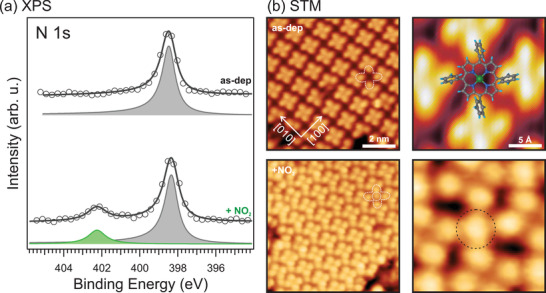
a) N 1s photoemission spectra, together with their corresponding fits, of the as‐deposited NiTPP/Cu(100) and after exposure to 10 L of NO_2_, bottom and top, respectively. 1 L = 1.33 × 10^‐6^ mbar × 1 s. The spectra are measured in a normal emission geometry at a photon energy of 1020 eV. b) STM images of NiTPP/Cu(100) before and after exposure to 10 L NO_2_ acquired at 77 K, top and bottom, respectively. Image size 8 × 8 nm^2^, tunneling parameters: as‐deposited *U* = −1 V, *I* = 500 pA, after the NO_2_ dose *U* = +1 V, *I* = 200 pA. On the right: close‐up of the corresponding NiTPP/Cu(100) interface before and after the NO_2_ dose. Image size 2 × 2 nm^2^, tunneling parameters: as‐deposited *U* = −1 V, *I* = 500 pA, after the NO_2_ dose *U* = +1 V, *I* = 200 pA.

At RT, both systems were exposed to increasing doses of NO_2_ and the resulting changes in the molecular film were monitored by means of XPS. The photoemission spectra of the N 1s core level shell before and after the exposure of the NiTPP/Cu(100) are shown in **Figure** **2**a, top and bottom, respectively. The N 1s spectrum of the pristine NiTPP on the copper surface shows a single peak at 398.6 ± 0.2 eV, as expected for the four chemically equivalent nitrogen atoms of the macrocycle.^[^ ^28^ ^]^ After exposing the Ni(I)TPP/Cu(100) interface to NO_2_, a new spectral feature appears at a binding energy of 402.5 ± 0.2 eV, while both the area and the BE of the pristine peak remain almost unaffected. Therefore, we tentatively assign this new component to the nitrogen atom of the NO_2_ ligand that adsorbs at the NiTPP/Cu(100) interface at RT.

To confirm the adsorption site of the ligand, STM measurements have been performed before and after dosing NO_2_ on the NiTPP layer. The corresponding STM images are shown in Figure 2b, top and bottom panels, respectively. The STM appearance of the as‐deposited NiTPP molecule is dominated by four bright features and a dark depression in the center (see Figure 2b, top right), associated with the four peripheral phenyl rings and the macrocycle core, respectively, as previously reported.^[^ ^24^ ^]^ After exposure to NO_2_, circular protrusions (circled in black) appear at the center of the NiTPP, replacing the depression at the macrocycle center (see Figure 2b, bottom right). From large‐scale STM images, it can be seen that such features appear on top of almost all the Ni sites (see Section S1, Supporting Information), and thus, in agreement with the XPS data, we can associate them with the NO_2_ molecule bound to the nickel ion. Crucially, we also observe that a gentle annealing up to 390 K is sufficient to restore the NiTPP film (see Section S1, Supporting Information).

Having established that NO_2_ interacts with the Ni(I)TPP molecule, we demonstrate that the +1 oxidation state is crucial for the film reactivity by performing a similar experiment on a Ni(II)TPP layer. Notably, in contrast to Ni(I)TPP, no change in the N 1s spectrum can be observed after dosing 20 L of NO_2_ on the Ni(II)TPP layer (see Section S2, Supporting Information). This suggests that the nickel is fully inert to NO_2_ in the higher (Ni(II)) oxidation state, concluding that the choice of a proper substrate, which can reduce the chelated nickel ion, is crucial for the reactivity of the molecular layer.

**Figure 3:**
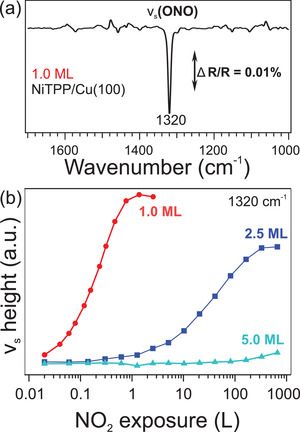
a) IRA spectra recorded after dosing NO_2_ onto 1.0 ML NiTPP/Cu(100) at 300 K. b) Peak height of ν_s_(ONO) as a function of the NO_2_ exposure for different NiTPP film thicknesses.

In the next experiment, we prove that the enhanced reactivity is a true interfacial property. For this, we deposited NiTPP films of different thicknesses (1.0, 2.5, and 5.0 monolayers [MLs]) atop Cu(100) and exposed them to different doses of NO_2_. After each NO_2_ uptake, we followed the changes at the interface by means of in situ IRAS, which allows us to access the interface despite the thickness of the molecular layer. A detailed analysis of the porphyrin vibrational modes in the as‐deposited single‐ and multi‐layer, as well as for the NO_2_‐NiTPP complex, agrees well with the data reported in our previous works (see Section S3, Supporting Information, for further details).^[^ ^29^, ^30^ ^]^ For the different NiTPP film thicknesses, the features that appear upon NO_2_ exposure are practically identical. However, the main difference between the three samples is the dose at which the NO_2_ signal grows. One feature related to the adsorbed NO_2_ is the peak at 1320 cm^−1^ which is associated with the symmetric stretching mode ν_s_(ONO) (see **Figure** **3**a). The peak height of this component is plotted as a function of the NO_2_ exposure in Figure 3b. We observe that for increasing film thickness, the required dose to form the adsorbed NO_2_ species increases drastically, while the intensity of the corresponding mode at saturation, decreases. These findings suggest that the NO_2_ diffuses through higher molecular layers and adsorbs on the Ni(I)TPP molecules of the first ML, while the Ni(II)TPPs, from the second layer on, are inert.

### 2.2 Spin Switching of the Central Ni Ion

**Figure 4:**
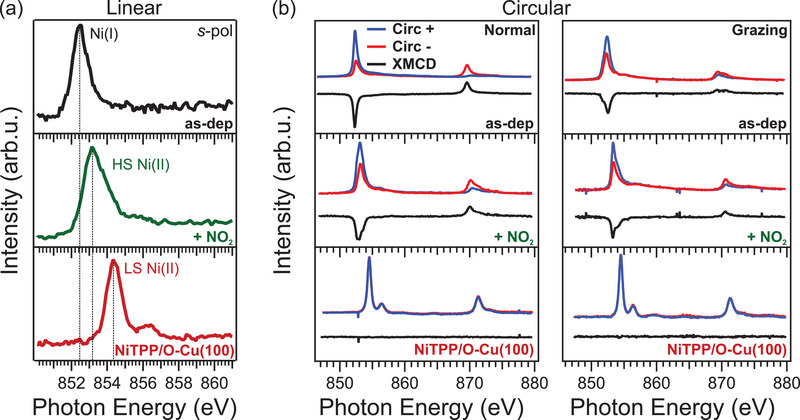
a) NEXAFS spectra at the L_3_‐edges of the three systems, namely, from top to bottom, NiTPP/Cu(100), NiTPP/Cu(100) after a dose of 10 L of NO_2_, and NiTPP/O–Cu(100). All the spectra were measured at room temperature, with linearly *s*‐polarized (*s*‐pol) light. b) Absorption spectra at the L_2,3_‐edge of left‐ and right‐hand circularly polarized light and resulting XMCD signals for normal (left) and grazing (right) incidence. Two different angles of incidence of the photon beam were used, that is, grazing (20° from the surface plane) and normal incidence, with an applied magnetic field B of 6.8 T aligned collinear to the beam propagation direction. The spectra were acquired at a temperature of 3 K. In order to ensure saturation of the NiTPP film, the system was exposed to 10 L of NO_2_.

We now quantitatively determine the electronic configuration of the central Ni ion in the three configurations considered so far (see Figure 1). To this end, we employed near‐edge X‐ray absorption fine structure (NEXAFS) measurements at the Ni L_3_‐edge to determine the oxidation state of the Ni ion. The corresponding results are reported in **Figure** **4**a. At the Ni L_3_‐edge, the excitations from occupied 2p core levels to unoccupied 3d valence states mostly contribute to the absorption spectrum of the incident light. Therefore, a change in the lineshape, as well as in the position of the absorption resonances, can provide information about the electronic structure of the Ni 3d shell.^[^ ^31^ ^]^

Considering first the pristine NiTPP film deposited on Cu(100), the centroid position of the main absorption resonance is at 852.4 eV (Figure 4a, top). This value is characteristic for the Ni(I) valence state and is associated with an intra‐atomic 2p_3/2_ → 3d_x2–y2_ transition.^[^ ^24^ ^]^ The single peak feature supports a d^9^ configuration with only one 3d hole.^[^ ^31^ ^]^ After exposing the Ni(I)TPP layer to NO_2_, the absorption spectrum changes drastically: the main peak shifts toward higher photon energies (853.2 eV) with a pronounced shoulder at 854 eV and a new broad resonance appears at about 856 eV (Figure 4a, middle). Both the lineshape and the peak position suggest a re‐oxidation of the nickel ion after coordination to the NO_2_ ligand, Ni(I) → Ni(II).^[^ ^31^ ^]^ The shoulder at higher photon energy is a characteristic marker of the high‐spin (HS) configuration of nickel‐containing complexes and metalloproteinase, where the Ni ion has two partially filled 3d orbitals.^[^ ^31^, ^32^, ^33^, ^34^ ^]^ These conclusions are further supported by the changes in the Ni 2p core‐level spectra upon NO_2_ exposure (see Section S5, Supporting Information).

On the other hand, the NEXAFS spectrum of the NiTPP/O–Cu(100) interface, instead, shows a sharp feature having its centroid at 854.3 eV, plus a small satellite peak at 856.5 eV (see Figure 4a, bottom). This confirms a low‐spin (LS) state for the Ni(II) in d^8^ configuration, with a completely unfilled orbital level $\left(d_{x^{2}-y^{2}}\right)$, which is typical of a tetra‐coordinated Ni(II),^[^ ^35^ ^]^ as in the gas‐phase porphyrin.

So far, NEXAFS measurements point toward three different spin configurations (d^9^, HS d^8^, and LS d^8^ for NiTPP/Cu(100), NO_2_‐NiTPP/Cu(100), and NiTPP/O–Cu(100), respectively). Since this assignment is based on a comparison between our NEXAFS spectra and reference data for the Ni ion,^[^ ^31^ ^]^ we performed additional X‐ray magnetic circular dichroism (XMCD) measurements on the three different interfaces to confirm this interpretation. The adsorption spectra acquired with left and right circularly polarized light at the L_3_‐ and L_2_‐edge are depicted in Figure 4b, together with the corresponding dichroic signal. In order to ensure magnetic saturation conditions, hysteresis curves were acquired in the [−6.8; 6.8]T range (see Section S6, Supporting Information).

While the NiTPP molecule deposited on the O–Cu(100) surface does not show any XMCD signal (see Figure 4b, bottom), thus confirming the gas‐phase LS d^8^ configuration, the situation is entirely different when the Ni porphyrin is in direct contact with the copper surface (see Figure 4b, top). Strong dichroism is present at the L_2,3_‐edge, pointing toward an HS configuration. By exposing the NiTPP/Cu(100) interface to NO_2_, we witness a further change in the XMCD (see Figure 4b, middle). The adsorption of NO_2_ does not quench the magnetic moment at the Ni center, supporting the scenario depicted above, that is, oxidation of the central metal ion from Ni(I) to Ni(II) followed by an HS transition. We also performed a sum rule analysis to obtain the effective spin moment^[^ ^36^ ^]^ and the orbital moment^[^ ^37^ ^]^ (see Section S7, Supporting Information, for further details). Measured in the two geometries in our experiment, normal, and grazing incidence, we found the effective spin moment (*m*_s,eff_) to be (1.64 ± 0.09)μ_B_ and (0.39 ± 0.05)μ_B_, respectively. Indeed, the values are similar to those of CuPc/Ag(100), where the Cu ion is also in a 3d^9^ configuration, that is, (1.67 ± 0.08)μ_B_ and (0.35 ± 0.08)μ_B_.^[^ ^38^ ^]^ Thus, we conclude that the values agree with the previously reported magnitude and anisotropy of the spin‐quadrupole coupling <T_z_>. For NO_2_‐NiTPP/Cu(100), the sum rule analysis yields an effective spin moment of (2.76 ± 0.2)μ_B_. As in an HS d^8^ and in a d^9^ configuration the electrons in the energetically highest orbitals do not carry orbital angular momentum (e_g_ orbitals), the values of the effective spin moment can, in a first approximation, be compared to the moments resulting from the spin‐only formula. Indeed, with values of 1.73 and 2.83 μ_B_ for one and two unpaired electrons, respectively, they lie within the error of our measurements.

From the sum rule analysis, it is possible to extract also the orbital magnetic moment (*m*_l_), which is responsible for the crystal‐field‐induced magnetic anisotropy of transition metal atoms. For the pristine molecular film, the out‐of‐the‐plane component is 275% higher than the in‐plane one (all the values for *m*_l_ and *m*_s,eff_ are reported in Table S1, Supporting Information). Although the *m*_l_ anisotropy does not reach the extremely high values around 490% found for CoOEP/graphene,^[^ ^39^ ^]^ it is higher than the one of the Fe–TPA_4_/Cu(100), which is around 190%^[^ ^6^ ^]^ and comparable with the one of the CuPc/Ag(100).^[^ ^38^ ^]^ After exposing NiTPP/Cu(100) interface to NO_2_, the out/in‐plane *m*_l_ ratio decreases to 162%. This behavior differs from that of similar systems, where an external ligand was adsorbed atop a coordinated metal ion. In those cases, the magnetic anisotropy is either almost quenched (CO–CoOEP/graphene^[^ ^39^ ^]^) or inverted (O_2_–Fe–TPA_4_/Cu(100)^[^ ^6^ ^]^). It is well known that the magnetic anisotropy determines the stability of the magnetization in the bulk as well as in nanoparticles.^[^ ^40^ ^]^ The fact that in the proposed interface, magnetic anisotropy does not change even in the presence of the external ligand, is of interest in the engineering of information storage devices at the atomic scale.

### 2.3 Theoretical Calculations

**Figure 5:**
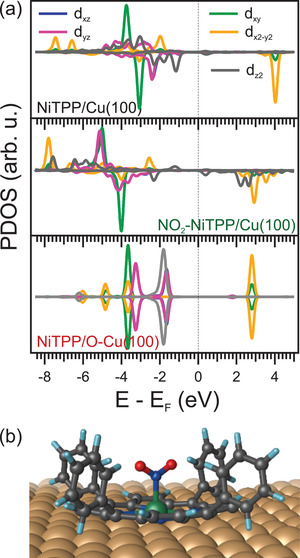
a) Calculated DOS projected onto the different d states of the central Ni atom for the majority and minority spin channels for the three interfaces, that are: NiTPP/Cu(100), NO_2_‐NiTPP/Cu(100), and NiTPP/O‐Cu(100). b) Proposed adsorption model for NO_2_‐NiTPP/Cu(100).

In order to support our experimental observations and provide a detailed insight into the observed on‐surface molecular spin‐switching, numerical simulations based on DFT were performed. The projected density of states (PDOS) onto the different 3d orbitals of the central Ni ion in each of the three systems under investigation is shown in **Figure** **5**a. For Ni(II)TPP on O–Cu(100) (see Figure 5a, bottom), the calculations confirm a gas phase‐like (d_xy_)^2^ (d_yz_, d_xz_)^4^ (d_z2_)^2^ (d_x2–y2_)^0^ configuration with two holes and no unpaired electrons. Consequently, the 3d orbital local magnetic density of states of Ni is equally distributed over the two spin‐channels (spin ↑ and ↓, respectively). This results in a vanishing magnetic dipole moment (*S* = 0) and is in agreement with the absence of XMCD signal.

In contrast, upon the interaction of NiTPP with a clean Cu surface, charge transfer across the interface leads to the occupation of formerly unoccupied molecular orbitals.^[^ ^24^ ^]^ This redistribution of electrons also enhances the electron density in the d_x2–y2_ nickel orbital, ultimately resulting in the oxidation of the central nickel atom with a (d_xy_)^2^ (d_yz_, d_xz_)^4^ (d_z2_)^2^ (d_x2–y2_)^1^ occupation (see Figure 5a, top). Nickel then has one unpaired electron, and the system becomes paramagnetic with *S* = 1/2. The net magnetic moment gives rise to a magnetic dichroic signal at the Ni L_2,3_‐edge (Figure 4b, top). Through NO_2_ coordination, the electronic structure of Ni changes once again, as revealed by the DOS projected onto the d‐states (see Figure 5a, middle). The d_z2_ states split and the spin ↓ part shifts above the Fermi level (*E*_F_). This corresponds, in first approximation, to a (d_xy_)^2^ (d_yz_, d_xz_)^4^ (d_z2_)^1^ (d_x2–y2_)^1^ configuration and, therefore, agrees well with the HS d^8^ configuration suggested by the NEXAFS analysis. Indeed, also in this case, a clear XMCD signal can be observed, as expected for two unpaired electrons.

### 2.4 The Macrocycle

**Figure 6:**
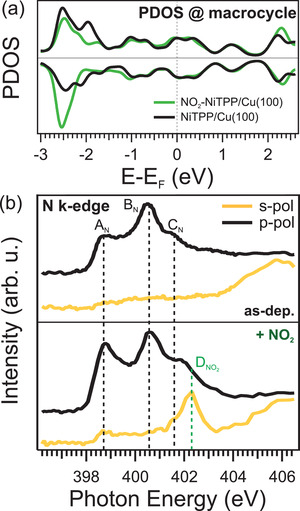
a) PDOS of the macrocycle for the NiTPP/Cu(100) and NO_2_‐NiTPP/Cu(100) systems. b) Nitrogen K‐edge absorption spectrum for the NiTPP/Cu(100) interface before (top) and after (bottom) the exposure of 10 L of NO_2_.

For the NO_2_‐NiTPP/Cu(100) system, the DFT optimized geometry shown in Figure 5b reveals that the NO_2_ ligand only slightly influences the NiTPP adsorption structure. Surprisingly, the ligand–porphyrin complex remains highly interacting with the Cu surface, as proven by the fact that neither the geometric parameters nor the DOS projected on the macrocycle (see **Figure** **6**a) are significantly altered by NO_2_ adsorption. As a consequence the massive charge transfer that populates the LUMOs, up to the LUMO+3, seems to be preserved. This is experimentally confirmed by the N K‐edge NEXAFS spectra taken with *s*‐ and *p*‐polarized light reported in Figure 6b. More specifically, the NEXAFS spectra of the Ni(I)TPP on Cu(100), acquired in *p*‐polarization, show three feature A_N_, B_N_, and C_N_ at 398.7, 400.6, and 401.6 eV, respectively. They originate from the π* resonances of the N 1s → LUMO/LUMO+1 and LUMO+3^[^ ^24^ ^]^ and exhibit strong dichroism, thus vanishing in *s*‐polarization spectra, which indicates a flat adsorption geometry of the porphyrin macrocycle. The strong interaction between the NiTPP and the Cu surface leads to the quenching of the resonance at 398.7 eV, whose intensity is reduced compared to the one observed for a molecular multilayer.^[^ ^24^ ^]^ Upon exposure of the porphyrin film to NO_2_, the NEXAFS spectrum measured with *s*‐polarized light shows a new feature at 402.3 eV with a shoulder at 401.5 eV (labeled D_NO2_, in Figure 6b, bottom), which we associated to the π* resonances of NO_2_.^[^ ^41^, ^42^ ^]^ The same peak is almost absent in *p*‐polarization, suggesting an upward geometry of the N—O bond in agreement with the calculated DFT adsorption structure and our IRAS results. Moreover, we also noticed only a moderate increase in the intensity of A_N_ and B_N_, which suggests that the high charge transfer at the NO_2_‐NiTPP/Cu(100) interface is almost preserved, in good agreement with the DFT predictions. As discussed in the previous section, the macrocycle backbone is seemingly unaffected by NO_2_ adsorption, thereby retaining its strong interaction with the Cu‐surface. This leaves the Ni atom as the main interaction partner for NO_2_. Our calculations reveal that NO_2_, which is acting as an additional ligand, increases the Ni–Cu surface distance by pulling the Ni atom ≈0.2 Å away from the surface toward the dioxide. The strong interaction of the porphyrin with the substrate seems to act as an energetic counterpart preventing further geometric changes induced by the so‐called surface *trans*‐effect. The latter is notably at work at other porphyrin/metal interfaces, where similar ligands with a strong donor/acceptor character, such as nitric oxide^[^ ^43^, ^44^ ^]^ and ammonia,^[^ ^17^, ^22^ ^]^ lift the whole molecule away from the substrate surface. Further details are given in Section S8, Supporting Information.

## 3 Conclusion

Altogether, the experimental and theoretical results presented here show that we can describe the NiTPP/Cu(100) interface in terms of two, mostly independent, subsystems: the central Ni ion and the macrocycle. On the one hand, the central Ni ion carries the spin moment that ultimately depends on its oxidation state and its chemical coordination to the surroundings. In the gas‐phase NiTPP molecule, Ni is in the Ni(II) oxidation state with *S* = 0. At the NiTPP/Cu interface instead, we observe Ni(I) with *S* = 1/2, (d_xy_)^2^ (d_yz_, d_xz_)^4^ (d_z2_)^2^ (d_x2–y2_)^1^, while when exposed to NO_2_, we observed a Ni(II) ion with even higher spin (*S* = 1), resulting from the electronic configuration (d_xy_)^2^ (d_yz_, d_xz_)^4^ (d_z2_)^1^ (d_x2–y2_)^1^. On the other hand, the frontier orbitals of the NiTPP, that are mainly located at the macrocycle, are stabilized in their electronic configuration by the charge transfer occurring from the copper surface, and do not react to the further bonding of the Ni ion to NO_2_. Moreover, the interface has proven to be extremely robust against thermal treatments^[^ ^30^ ^]^ and a further molecular deposition does not substantially alter the reactivity of the film.

In the past, porphyrin molecules have been proposed as new class of multifunctional materials.^[^ ^45^ ^]^ In that context, however, the term “multifunctional” was referred to the possibility of employing them in an extremely wide range of applications rather than to simultaneously control different molecular properties at once. In the last 15 years, spintronics has successfully pursued the latter strategy, resulting in the design of the first prototypes of multipurpose spintronic devices. Among others, photoresponsive molecular spintronic devices^[^ ^46^ ^]^ and molecular spin photovoltaic devices based on fullerene^[^ ^47^ ^]^ have been realized. The advances in the field have been reviewed by L. Guo et al.^[^ ^48^ ^]^ only recently. In this perspective, our work provides a microscopic picture about the fundamental mechanisms involved in the spin and electronic functionality and paves the way to exploit the full potential of porphyrin‐based devices. More specifically, by the unique combination of the Ni metallic center (chelated in a tetraphenyl porphyrin) and the copper substrate, we established a suitable platform for spin‐switching applications via a chemical stimulus, where the Ni functionalization by means of NO_2_ does not alter the Ohmic contact between the porphyrin macrocycle and the surface underneath. To our knowledge, this system constitutes the first example of a RT on‐spin‐switch with stable magnetic anisotropy that preserves its properties even in the buried interface, making it appealing for more applicative studies.

As an example, we envision a simple spintronic building block based on a very thin copper layer covered by NiTPP molecules. If placed in an external magnetic field, the resistance of the device will change when it is exposed to NO_2_, as a result of the modification in the spin‐dependent scattering of the carriers with the magnetic moments of the central Ni atom (interfacial magneto‐resistance effect). We expect that this device could be used as a very sensitive NO_2_ detector, but also as a building block for performing spin memory and logic operations triggered by the presence of NO_2_.

## 4 Experimental Section

### Sample Preparation

The Cu(100) surface was prepared by several cycles of sputtering Ar^+^ at 2.0 keV and subsequent annealing of the sample up to 800 K. NiTPP molecules (Sigma Aldrich, purity 95%) were thermally sublimated at 570 K from a Knudsen cell type evaporator onto the copper substrate kept at RT. One ML corresponds to a densely packed, single‐layer molecular film. At the ALOISA beamline, the evaporation rate was checked with a quartz microbalance. The oxygen reconstructed surface was prepared by dosing 800 L of O_2_ while keeping the substrate constantly at 500 K,^[^ ^49^ ^]^ and the (√2 × 2√2)R45°reconstruction was checked by LEED and STM.

### X‐Ray Magnetic Circular Dichroism

The difference between two absorption spectra measured with circularly polarized X‐ray light with opposite helicities (µ_+_ and µ_−_) was evaluated at the L_3_ and L_2_ absorption edges in order to check for a spin‐polarization of the empty states. The normalization by the isotropic XAS spectrum was done by dividing through the sum of the integrated absorption intensities of both helicities. The measurements were carried out at the EPFL/PSI X‐treme beamline, at the Swiss Light Source, Paul Scherrer Institute, Villigen, Switzerland.^[^ ^50^ ^]^ They were performed in a total electron yield mode, measuring the drain current of the sample. A magnetic field of 6.8 T was applied parallel to the light propagation in order to maximize the signal, and the sample was rotated in order to measure the absorption with normal (Θ = 0°) and grazing (Θ = 70°) incidence. The sample temperature for all the spectra presented here was around 3 K.

### Near‐Edge X‐Ray Absorption Fine Structure and X‐Ray Photoelectron Spectroscopy

The NEXAFS and X‐ray photoemission spectra were measured at the ALOISA beamline^[^ ^51^ ^]^ of the Elettra Synchrotron in Trieste, Italy. The N 1s spectra were fitted using a multipeak Voigt function. The N 1s FWHM of the porphyrin‐ and NO_2_‐related peaks were 0.8 and 1.5 eV, respectively. The Ni 2p spectra, instead, were analyzed by exploiting a Doniach‐Šunjić lineprofile, convoluted with a Gaussian to account for energy resolution and thermal and inhomogeneity broadening after the subtraction of a Shirley‐type background. For both the N 1s and Ni 2p spectra, a Gaussian broadening of 300 meV was assumed. At ALOISA, the sample was mounted at grazing incidence on a manipulator coaxial to the photon beam. The XPS spectra were measured at a grazing angle of 4° in transverse magnetic polarization (quasi *p*‐pol) with the electron spectrometer in normal emission. The total electron energy resolution (analyzer and beamline) was set to 300 meV. The NEXAFS spectra were measured in a partial electron yield mode using a Channeltron multiplier equipped with a front grid polarized at negative bias to reject low energy secondary electrons (−820 V for the Co L_2_‐edge and −370 V for the N K‐edge). The polarization was changed from *p*‐pol to transverse electric (*s*‐pol) by rotating the sample around the photon beam axis, while keeping the sample at constant grazing angle (and illuminated area) of 6°.

### Scanning Tunneling Microscopy

Low‐temperature STM experiments were carried out with an Omicron LT‐STM system located at CNR‐IOM, Trieste, Italy. All the measurements were performed at a sample temperature of 77 K. The microscope was hosted in an ultrahigh vacuum (UHV) chamber, operating at a base pressure below 7 × 10^−11^ mbar. Images were acquired in constant current mode. Electrochemically etched tungsten tips were used for imaging. The sample was prepared in a separate chamber with a base pressure of 2 × 10^−10^ mbar, connected to the STM chamber. At each annealing step, the sample was kept at the corresponding temperature for 5 min and then cooled down to 77 K in the STM before the measurement.

### Infrared Reflection Absorption Spectroscopy

The IRAS experiments were performed in a UHV chamber with a base pressure of 1 × 10^−10^ mbar. The apparatus was equipped with various preparation and characterization tools described in further detail elsewhere.^[^ ^52^ ^]^ A remote‐controlled gas dosing system and evaporation sources allow for in situ investigation with a vacuum Fourier‐transform infrared (FT‐IR) spectrometer (Bruker Vertex 80v).

During evaporation of NiTPP at 570 K, time‐resolved IRA spectra were recorded at a rate of 1 spectrum/min and a spectral resolution of 4 cm^−1^. The spectra were referenced to a background spectrum recorded with an acquisition time of 10 min on the clean Cu(100). A new background spectrum (acquisition time 10 min) was acquired on the NiTPP film, serving as a reference for the NO_2_ adsorption experiments. Via the remote‐controlled gas dosing system, different pulses of NO_2_ (Linde, 2.0) were applied to the sample at a pressure of 1 × 10^−6^ mbar. An IR spectrum with a spectral resolution of 4 cm^−1^ (acquisition time 5 min) was recorded after each gas dose.

The attenuated total reflection (ATR) IR reference spectrum of NiTPP (acquisition time 120 s [256 scans], spectral resolution 2 cm^−1^) was measured using a FT‐IR spectrometer (Bruker Vertex 80v) with a hemispherical Ge ATR window (Bruker).

### Computational Methods

All calculations were performed within the framework of DFT using the Vienna Ab Initio Simulation Package version 5.4.4.^[^ ^53^, ^54^ ^]^ Exchange‐correlation effects were described by the functional of Perdew–Burke–Ernzerhof^[^ ^55^ ^]^ and van der Waals contributions treated with the D3 dispersion correction.^[^ ^56^ ^]^ The projector augmented wave method^[^ ^57^ ^]^ was utilized together with an energy cutoff of 400 eV. All calculations were spin‐polarized with the ionic positions of all structures optimized within 10^−6^ eV. The surfaces were simulated within the repeated slab approach using four substrate layers, two of which were held‐fix during optimization, and a 25 Å vacuum layer. To avoid spurious electrical fields, a dipole layer was inserted in the vacuum region.^[^ ^58^ ^]^ The results were obtained on a Monkhorst–Pack^[^ ^59^ ^]^ 4 × 4 × 1 grid of k‐points and a Methfessel–Paxton smearing of first order of 0.1 eV. Subsequent to the geometry relaxation, the DOS were calculated non‐self consistently using the Heyd–Scuseria–Ernzerhof^[^ ^60^ ^]^ short‐range hybrid functional to account for the presence of both the delocalized π‐orbitals of the macrocycle and the localized states at the Ni atom.

## Conflict of Interest

The authors declare no conflict of interest.

## Author Contribution

The project was conceived by G.Z. and V.F. together with M.C. and C.M.S. H.M.S., I.C., G.Z., and V.F. carried out the photoemission and XMCD and NEXAFS experiments and the consequent data analysis with support from E.V., M.S., C.P., M.J., A.V., A.C., and L.F. P.P and A.W. performed the DFT simulations. A.S., C.A., and G.C. carried out the STM measurements and M.K., C.H., and J.L. performed in situ IRAS experiments and the consequent data analysis. G.Z. together with H.M.S., M.C., I.C., and V.F. prepared the draft and all authors discussed the results and reviewed the manuscript.

## Supporting information

Supporting Information

## Data Availability

The authors declare that relevant data supporting the findings of this study are available upon reasonable request.
