# Supplementary material for: Room‐Temperature On‐Spin‐Switching and Tuning in a Porphyrin‐Based Multifunctional Interface
Source: Small. 2021 Oct 12;17(50):2104779. doi: 10.1002/smll.202104779 (PMC11475682; doi:10.1002/smll.202104779)
Supplement: Supplementary file 1 — Supporting Information [file SMLL-17-2104779-s001.pdf]

NANO small MICRO

Supporting Information

for *Small,* DOI: 10.1002/smll.202104779

Room-Temperature On-Spin-Switching and Tuning in a Porphyrin-Based Multifunctional Interface

Henning Maximilian Sturmeit, Iulia Cojocariu, Andreas Windischbacher, Peter Puschnig, Cinthia Piamonteze, Matteo Jugovac, Alessandro Sala, Cristina Africh, Giovanni Comelli, Albano Cossaro, Alberto Verdini, Luca Floreano, Matus Stredansky, Erik Vesselli, Chantal Hohner, Miroslav Kettner, Jörg Libuda, Claus Michael Schneider, Giovanni Zamborlini, Mirko Cinchetti, and Vitaliy Feyer*

Supporting Information

Room-temperature on-spin-switching and tuning in a porphyrin-based multifunctional interface.

Henning Maximilian Sturmeit, Iulia Cojocariu, Andreas Windischbacher, Prof. Peter Puschnig, Dr. Cinthia Piamonteze, Dr. Matteo Jugovac, Dr. Alessandro Sala, Dr. Cristina Africh, Prof. Giovanni Comelli, Prof. Albano Cossaro, Dr. Alberto Verdini, Dr. Luca Floreano, Dr. Matus Stredansky, Prof. Erik Vesselli, Chantal Hohner, Dr. Miroslav Kettner, Prof. Jörg Libuda, Prof. Claus Michael Schneider, Dr. Giovanni Zamborlini, Prof. Mirko Cinchetti, Dr. Vitaliy Feyer

SI 1 – STM, large scale images and reversibility of NO_2 binding

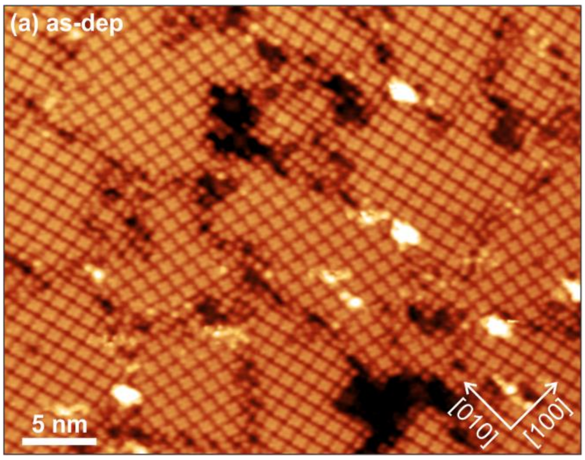

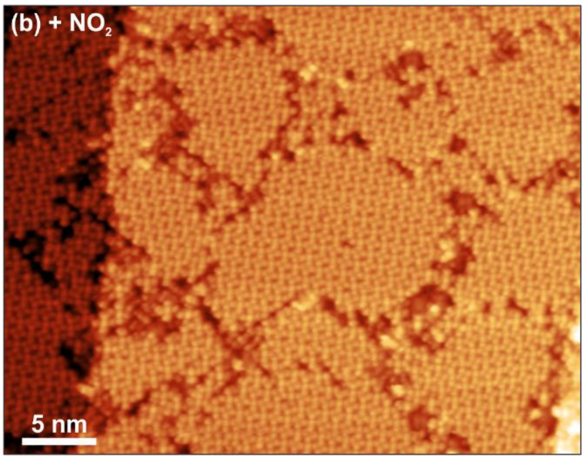

Figure S1. Large-scale STM images of NiTPP/Cu(100) before (a) and after (b) exposure to 10 L NO_2 acquired at 77 K. Image size 38.8 x 30 nm^2. Tunnelling parameters: (a) U=+1 V, I=500 pA, (b) V_b=+1 V, I=200 pA.

Large-scale STM images of the as-deposited NiTPP film on Cu(100) depicted in **Figure S1a** confirm the presence of the two mirrored domains rotated by about ± 8° with respect to the [001] direction of the Cu substrate, as already reported in Ref.[1] They can be described by the following epitaxial matrices (4,3/-3,4) and (3,4/-4,3). Upon NO_2 exposure, the dark depression at the macrocycle centre is replaced by a bright protrusion associated with the nitrogen dioxide (as described in the main text). The corresponding large-scale image is shown in **Figure S1b**. We note that almost all the Ni centres are now bright, indicating that the NiTPP array is saturated with NO_2. Moreover, we see no change neither in the unit cell nor in the molecular orientation.

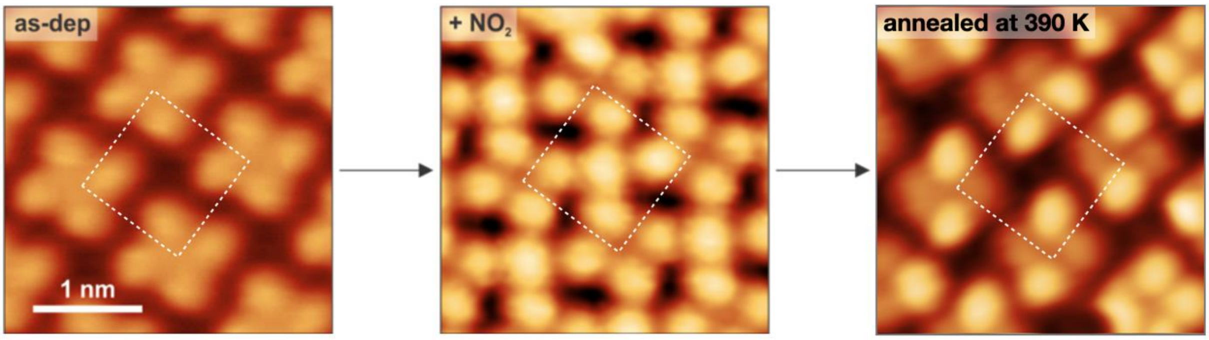

Figure S2. STM images of NiTPP/Cu(100) before (left) and after (middle) exposure to 10 L NO_2 and after annealing (right) up to 390 K. A single NiTPP molecule is framed with a dashed white square to guide the eye. All images are acquired at 77 K. Image size 3x3 nm^2. Tunnelling parameters: (left) V_b=-1 V, I=500 pA, (centre) V_b=+1 V, I=200 pA, (right) V_b=-1 V, I=200 pA.

In a next step, we show that the adsorption process is reversible and thus NO_2 can be desorbed from to the Ni centre by annealing the system at 390 K. The corresponding changes in the NO_2-NiTPP/Cu(100) layer were monitored by STM and the images are reported in **Figure S2**. As shown in the main text, the as-deposited NiTPP molecules (left) present a rounded protrusion at the centre after exposure to 10 L of NO_2 (middle). After the annealing at 390 K (right) all NO_2 molecules are desorbed from the NiTPP molecules, restoring the dark depression at the macrocycle centre, while the appearance of the lateral features related to the phenyl rings drastically changes. This phenomenon has been previously observed and it is associated to a flattening of the phenyl moiety upon sample annealing.[2] The flattening is in general not reversible, as the thermal treatment allows the molecule to reach a more stable adsorption configuration. Notably, in order to have a uniform NiTPP layer with all the phenyls in the final geometry, higher annealing temperatures, *i.e.* 470 K, are necessary.[2]

SI 2 – N 1*s* XPS of NiTPP/O-Cu(100)

The chemical reactivity of the Ni ion in dependence of its oxidation state can be tested by exposing the NiTPP/O-Cu(100) film to NO_2. Indeed, the covalent nature of the Cu–O interaction yields a strong localization of the surface electrons, inhibiting the charge transfer

from the substrate to the organic film[3,4], preserving the Ni(II) oxidation state of the gas-phase NiTPP.

As depicted in **Figure S3**, after dosing 20 L of NO_2 neither a second feature nor a broadening could be observed in the N 1*s* XPS spectrum, indicating that the NO_2 does not bind to the

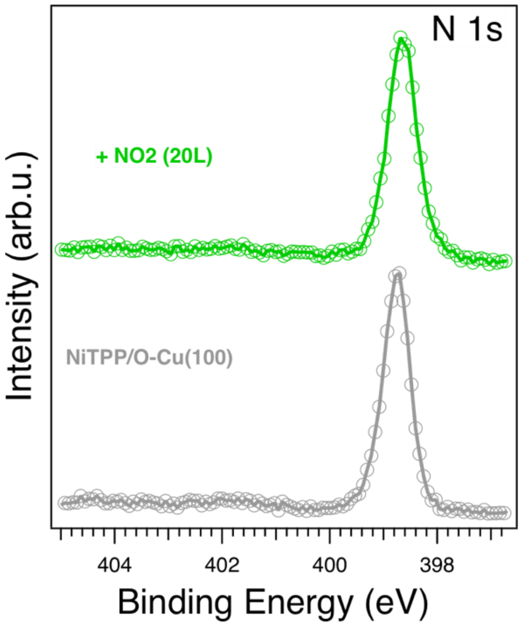

Figure S3. N 1*s* spectrum of NiTPP/O-Cu(100) before and after dosing 20 L of NO_2. The spectra are measured in a normal emission geometry at a photon energy of 515 eV.

NiTPP array.

SI 3 – IRAS measurements of the NiTPP/Cu(100) interface

To characterize the interaction of NiTPP with Cu(100) and the adsorption behaviour of NO_2, we performed IRAS experiments under UHV conditions. First, we monitored the deposition of a NiTPP multilayer (roughly 5 ML) on clean Cu(100) at 300 K *in-situ* by IRAS. In **Figure S4** we show selected IRA spectra recorded during the uptake at different NiTPP coverages. In the multilayer spectra, the main bands appear at 3062, 3025, 1600, 1442, 1353, 1073, 1008, 800 and 752 cm^{-1}. According to the literature, we assigned the signals to specific modes which are located predominately either at the central porphine macrocycle or at the phenyl rings[5–10]. The ATR reference spectrum of NiTPP is in good agreement with the multilayer IRA spectrum, confirming that the porphyrin is deposited without decomposition.

In the monolayer (ML) regime (marked in blue) most of the multilayer signals are present and have the same shape. However, there are some missing features, namely the bands at 3025, 1353 and 1008 cm^{-1} (marked in grey), which we ascribe to C-H stretching ν(C-H), combined stretching of C-C, C-N (and C-H) in the pyrrole units $\nu(\text{C-N, C-C, (C-H)})_{porphine}$, and in-plane

Figure S4. Top: Selected IRA spectra recorded during deposition of 5 ML NiTPP on Cu(100) at 300 K (ip = in plane, oop = out of plane). Bottom: Reference ATR IR spectrum of NiTPP.

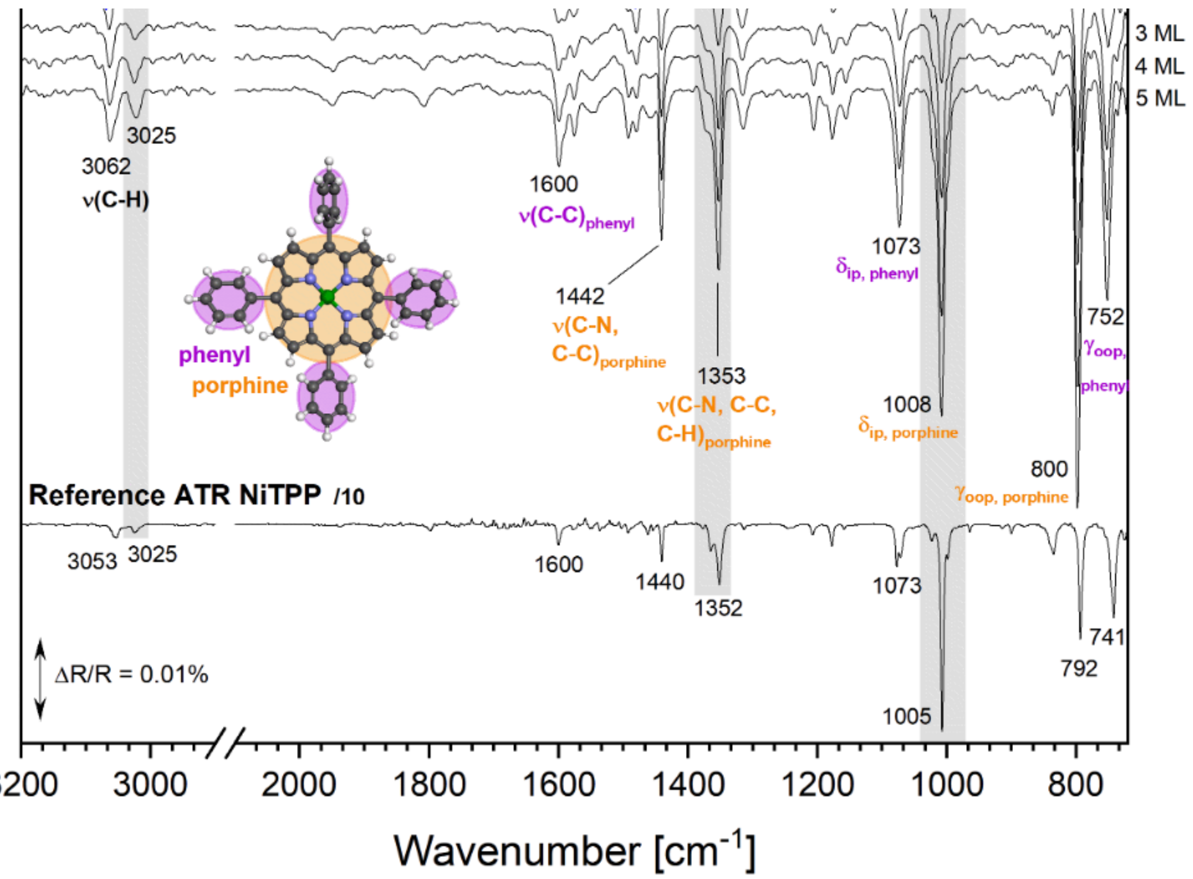

deformation of the porphine $\delta_{ip,porphine}$. Since IRAS is sensitive to the adsorption geometry of adsorbates, changes between monolayer and multilayer spectra often indicate distinct orientations of the adsorbates near the surface. The reason is the metal surface selection rule (MSSR), which implies that only those modes are active in IRAS which have a dynamic dipole moment that is not parallel to the surface.[11] The vibration at 1008 cm^{-1}, for example, resembles deformations within the molecular plane and is not present in the monolayer spectrum. Thus, we conclude that the corresponding dynamic dipole moment is oriented largely parallel to the metal surface (see below for further details about the IR activity). This finding implies that the NiTPP is lying flat on the Cu(100) surface, in agreement with the adsorption geometries of (M)TPP reported in the literature[12–16] and with the DFT calculations in this work. As soon as the multilayer forms, the three missing signals rapidly gain intensity, which is in line with the loss of preferential orientation. These characteristic differences between the monolayer and multilayer spectra allow to estimate the porphyrin coverage directly from the IR spectra.

In contrast to classical IR spectroscopy, the dynamic dipole of a vibration must fulfill an additional selection rule for IRAS metals, namely the metal surface selection rule (MSSR). The MSSR states that components of the dynamic dipole moment which are parallel to the metal surface do not give rise to an absorption band. We now consider four IRA signal components of the NiTPP monolayer on Cu(100): the in-plane deformation vibrations of the porphine $\delta_{ip,\ porphine}$ (1008 cm^{-1}) and phenyl unit $\delta_{ip,\ phenyl}$ (1073 cm^{-1}) and the out-of-plane deformation modes of the porphine $\gamma_{oop,\ porphine}$ (800 cm^{-1}) and phenyl unit $\gamma_{oop,\ phenyl}$ (752 cm^{-1}). Since the flat lying NiTPP has a molecular plane parallel to the underlying substrate surface, the porphine in-plane vibration is also parallel and thus IR inactive, whereas the out-

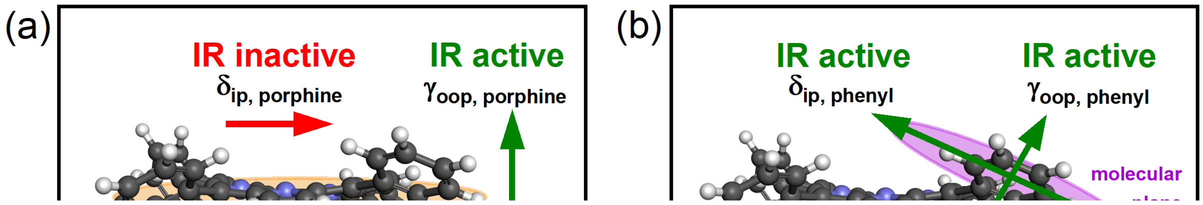

Figure S5. Model of the adsorbed NiTPP, orientation of the dynamic dipole moment for (a) $\delta_{ip,\ porphine}$ and $\gamma_{oop,\ porphine}$, (b) $\delta_{ip,\ phenyl}$ and $\gamma_{oop,\ phenyl}$ (ip = in plane, oop = out of plane).

of-plane vibration is strongly IR active (**Figure S5a**). Both deformation vibrations of the phenyl groups feature dynamic dipole components parallel and orthogonal to the surface. Thus, they are IR active but less intense due to the loss of the parallel component (**Figure S5b**).

SI 4 – NO2 uptake as function of NiTPP thickness

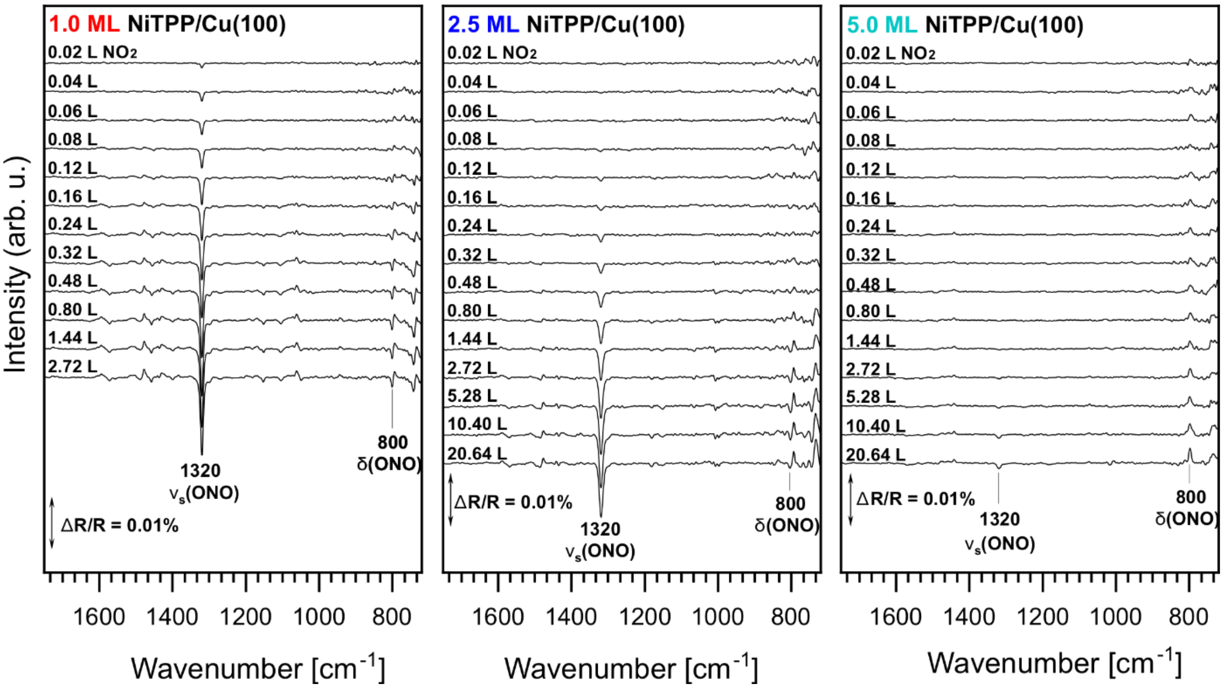

Figure S6. IRA spectra of the NO_2 dosing on NiTPP/Cu(100) at 300 K for different NiTPP film thicknesses (1.0, 2.5 and 5.0 ML). All the IR spectra are referenced to the background acquired on the as-prepared NiTPP. Thus, exclusively the changes are visible which occur upon NO2 exposure. The main vibrational features related to the NO_2 adsorption are described in the main text.

During the NO_2 uptake, all IR spectra are referenced to the background acquired on the as-prepared NiTPP. Thus, we observe only the changes which occur upon NO_2 exposure. We observe in **Figure S6** the appearance of two signal components at 1320 and 800 cm^{-1} for the 1 ML NiTPP sample. According to the literature, NO_2 typically features three main vibrational fingerprints: an asymmetric O-N-O stretching band ν_{as}(ONO) in the 1600-1750 cm^{-1} range, a symmetric O-N-O stretching ν_s(ONO) in the 1200-1300 cm^{-1} and O-N-O bending δ(ONO) in the 750-800 cm^{-1} intervall.[17–20] Thus, we assign our signals to the symmetric stretching mode

ν_s(ONO) and at 800 cm^{-1} to the bending mode δ(ONO), respectively. However, we do not observe the asymmetric O-N-O stretching vibration. An explanation for the absence of this mode is the molecular orientation of the adsorbed NO_2 that creates a dynamic dipole which is completely parallel to the surface and, therefore, forbidden by the metal surface selection rule (MSSR).[21]

Based on all the above observations, we propose an adsorption geometry where NO_2 binds to the Ni center through the central nitrogen atom. Here, the two oxygen atoms point upwards in a "V" shape configuration, leading indeed to an IR active ν_s(ONO) mode and to an IR inactive ν_{as}(ONO) mode (see **Figure S7**).

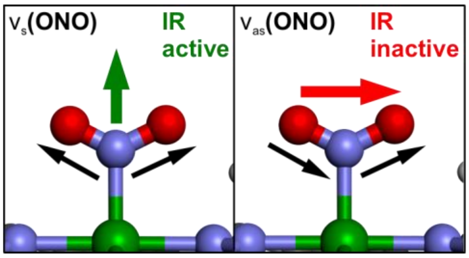

Figure S7. Schematic representation of the IR active and inactive ν_s(ONO) and ν_{as}(ONO) mode.

Because of the fast (compared to the STM acquisition time) precession motion of nitrogen dioxide, this adsorption configuration gives rise to a time-averaged disc-shaped appearance of the NO_2 molecule in the STM topographic images instead of protrusion in form two lobes.[22]

In case of the multilayer samples there are weak features at 800 cm^{-1} upon NO_2 exposure, which have an s-shape or are pointing upwards. We attribute these features to bands of the NiTPP which experience small shifts as a result of reorientation or changes in intermolecular interaction.

SI 5 – Ni 2*p* XPS

Upon exposure of the NiTPP/Cu(100) interface to 15 L of NO_2, a new feature appears in the N 1s, as described in the main text. We now focus on the changes occurring at the Ni $2p_{3/2}$ core level. For the pristine molecular layer, it is composed of a single peak at a binding energy of 852.9 ± 0.2 eV, which is specific for the Ni(I) ion (**Figure S8**, bottom), as previously reported.[23] After the exposure of the NiTPP/Cu(100) to NO_2, a new feature appears at a BE of 854.6 ± 0.2 eV, while the Ni(I) component decreases substantially (Figure S8, top). This suggests that NO_2 binds directly to the Ni ion and the saturation of the metal porphyrin centre is almost complete. The BE shift in the Ni $2p_{3/2}$ is associated with oxidation of Ni ion upon NO_2 coordination, in agreement with NEXAFS data measured across Ni L-edge.

By combining the information provided by both N 1*s* and Ni 2*p* core levels, a semi-quantitative analysis can be carried out to exclude a multiple NO_2 adsorption at the same Ni site. Taking into account that every porphyrin has four nitrogen atoms and not all the NiTPP molecules host the external ligand, the areas between the two N 1*s* components, one

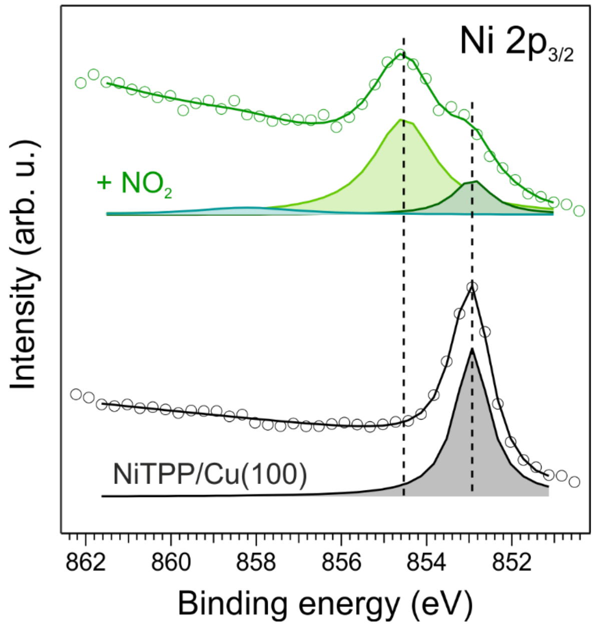

Figure S8. Ni 2*p* photoemission spectra, together with their corresponding fits, of NiTPP/Cu(100) before and after exposure to 10 L of NO_2. The spectra are measured in a normal emission geometry at a photon energy of 1020 eV.

associated to the N atoms of the porphyrin and the other to the NO_2, can be compared. In doing so, we neglect photoelectron diffraction effects and assume a constant transmission of the analyser at the corresponding kinetic energies. This yields a ratio close to 1:4 between the two peaks, suggesting that one single NO_2 molecule binds at the Ni centre of every NiTPP.

SI 6 – Hysteresis curve

To check for magnetic saturation, the XMCD intensity at the L_3 edge was measured as a function of the applied magnetic field in the range between -6.8 T and 6.8 T. The measurements are done by acquiring the TEY as a function of the applied magnetic field, at the energy of maximum XMCD and at the pre-edge where there is no XMCD contrast, in order to account for baseline drifts. The same measurement is repeated for different x-ray helicities in order to obtain the XMCD magnitude as a function of the applied field.

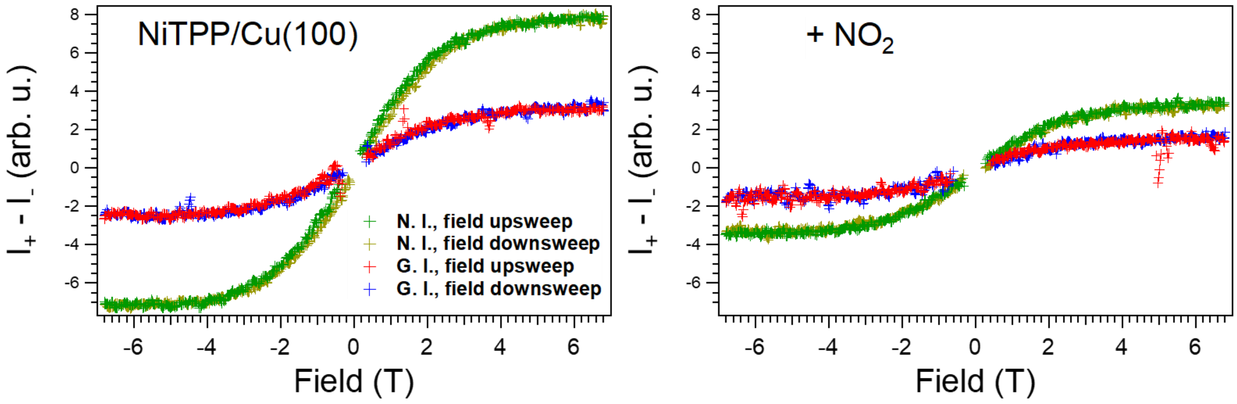

Figure S9. Magnetic hysteresis curves for NiTPP/Cu(100) before (bottom panels) and after (top panels) dosing 10 L NO_2 measured at 2 K. For both systems a magnetic saturation is reached for normal (N.I., left panels) and grazing (G.I., right panels) photon incidence, respectively.

SI 7 – Sum rule analysis

The calculated values of the sum rule analysis are shown in **Table S1**. Since the charge transfer at the NiTPP/Cu(100) interface leads to a change of the nickel oxidation state, we applied the same normalization for the isotropic absorption intensity over both edges as in Ref.[24]

	Normal incidence (out-of-the-plane **OP**)	Grazing incidence (out-of-the-plane **IP**)	g (OP)	g (IP)
**NiTPP/Cu(100)**				
$m_{s,eff}$	$(1.64 \pm 0.09)\mu_B$	$(0.39 \pm 0.05)\mu_B$	2.44	2.16
m_l	$(0.22 \pm 0.02)\mu_B$	$(0.08 \pm 0.01)\mu_B$		
**NO_2-NiTPP/Cu(100)**				
$m_{s,eff}$	$(2.76 \pm 0.2)\mu_B$	$(0.73 \pm 0.05)\mu_B$	2.29	2.18
m_l	$(0.29 \pm 0.05)\mu_B$	$(0.18 \pm 0.01)\mu_B$		

Table S1. Resulting values for the effective spin moments, orbital moments and g values of NiTPP/Cu(100) and NO_2-NiTPP/Cu(100) for normal and grazing incidence, respectively.

As can be expected from a similar ligand field and a $3d^9$ configuration, when going from out-of-plane towards in-plane direction, i.e. 70° off normal, a decrease of the orbital magnetic moment decreasing from $(0.22 \pm 0.02)\mu_B$ to $(0.15 \pm 0.02)\mu_B$ could be observed, even though not as severe as for CuPc/Ag(100).[24]

Another way to appreaciate the anisotropy of the system is to calculate the gyromagnetic factor (g) via the following equation:

$$g = 2 \cdot \left(1 + \frac{\mathrm{m}_L}{m_S}\right)$$

The resulting values are reported in the Table S1and are then compared with the ones measured for similar systems (reported in Table S2). Also from the g factor, it is clear that both the easy magnetization axis of the NiTPP/Cu(100) and NO_2-NiTPP/Cu(100) lay out-of-the-plane, but upon functionalization the anisotropy of the system is reduced.

	g (out-of-the-plane)	g (in-plane)	ratio (OP/IP)
**Hermanns *et al*.** *PRB* **88**, 104420 (2013)			

CoOEP/Graphene	2.38	3.6	0.66
**S. Stepanow *et al.*** *PRB* **82**, 014405 (2010)			
CuPC/Ag(100)	2.33	2.13	1.09

Table S2. g factor of CoOEP/Graphene and CuPC/Ag(100) for the out-of-the-plane and in-plane componets.

We also note that anosotropy of the *g* factor is consistent with values reported for comparable molecular-based interfaces.

SI 8 – DFT

As the DFT calculations have proven to be valuable in predicting the oxidation state and spin change of the Ni atom between the three investigated systems, they may also provide further insight into the chemical nature of the interfaces. As could be expected from the previously reported absence of the LUMO/+1 and LUMO+3 features in the valence band photoemission spectrum[3], the molecule shows gas phase-like behavior on the oxygen passivated Cu-surface. Here, it is important to note that also the calculated bond lengths of pyrrolic C-C and C-N bonds are typical of the conjugated porphyrin macrocycle (~1.39 Å for C-N bonds)[25,26] and no significant hybridization of the surface with the electronic levels of the molecule takes place. In contrast, when turning to the bare Cu-surface, we observe strong interaction between the surface and NiTPP.[1,2] Apart from geometrical changes (*i.e.* twist deformations of the phenyls to maximize the contact area of the macrocycle with the substrate), the interaction with the Cu substrate also results in the elongation of the bonds of the conjugated porphyrin system towards a single bond character (~1.44 Å). This geometric change is a direct consequence of the increased electron density after charge transfer to formerly unoccupied states with antibonding π* character, which are predominantly located at the macrocycle, thus reducing the formal bond order.

The fact that the charge transfer at porphyrin/copper interfaces is not quenched upon NO_2 adsorption, further suggests that the switch of the spin state at the metal strongly relates to the gas uptake. As a test for this hypothesis, we have computed the NO_2-NiTPP complex on O-Cu(100), where again the passivated surface causes gas-phase-like behavior. Note that the only purpose of this calculation is to understand the role of the exceptionally strong

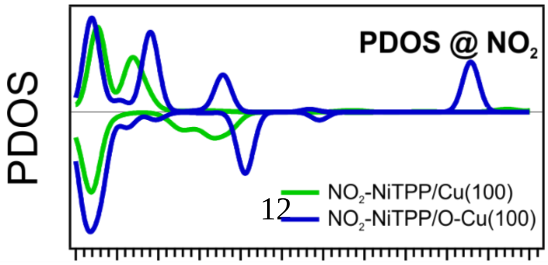

Figure S10. PDOS of the NO_2 molecule for NiTPP/Cu(100) and NO_2-NiTPP/Cu(100) systems.

porphyrin-substrate interaction at the NiTPP/Cu(100) interface and that there was no NO_2 uptake of the NiTPP on the passivated surface observed at room temperature. In Figure S10, we compare the DOS projected onto the NO_2 ligand of the complex on both surfaces. As expected, we see a significant contribution above E_F in only one spin channel for NO_2-NiTPP on O-Cu(100), which accounts for the radical nature of NO_2. In contrast, this contribution is gone on the bare Cu surface. Analyzing the local magnetic moments, we notice that indeed any paramagnetic contributions come solely from the Nickel atom. Thus, we conclude that there is an electron uptake by the NO_2, accompanied by oxidation of the nickel ion ($d^9 \rightarrow d^8$). Moreover, the additional electron density at the NO_2 ligand destabilizes the Ni d_{z2} orbital to such an extent that the orbital splitting reduces sufficiently to form a high spin complex.[27,28]

Supporting references

[1] G. Zamborlini, D. Lüftner, Z. Feng, B. Kollmann, P. Puschnig, C. Dri, M. Panighel, G. Di Santo, A. Goldoni, G. Comelli, M. Jugovac, V. Feyer, C. M. Schneidery, *Nat. Commun.* **2017**, *8*, 1.

[2] H. M. Sturmeit, I. Cojocariu, M. Jugovac, A. Cossaro, A. Verdini, L. Floreano, A. Sala, G. Comelli, S. Moro, M. Stredansky, M. Corva, E. Vesselli, P. Puschnig, C. M. Schneider, V. Feyer, G. Zamborlini, M. Cinchetti, *J. Mater. Chem. C* **2020**, *8*, 8876.

[3] I. Cojocariu, H. Maximilian, G. Zamborlini, A. Cossaro, A. Verdini, L. Floreano, E. D. Incecco, M. Stredansky, E. Vesselli, F. J. Gmbh, *Appl. Surf. Sci.* **2020**, *504*, 144343.

[4] X. Yang, I. Krieger, D. Lüftner, S. Weiß, T. Heepenstrick, M. Hollerer, P. Hurdax, G. Koller, M. Sokolowski, P. Puschnig, M. G. Ramsey, F. S. Tautz, S. Soubatch, *Chem. Commun.* **2018**, *54*, 9039.

[5] K. Werner, S. Mohr, M. Schwarz, T. Xu, M. Amende, T. Döpper, A. Görling, J. Libuda, *J. Phys. Chem. Lett.* **2016**, *7*, 555.

[6] X. Y. Li, R. S. Czernuszewicz, J. R. Kincaid, Y. O. Su, T. G. Spiro, *J. Phys. Chem.* **1990**, *94*, 31.

[7] S. Mishra, S. Kaur, S. K. Tripathi, C. G. Mahajan, G. S. S. Saini, *J. Chem. Sci.* **2006**, *118*, 361.

[8] T. Wähler, R. Schuster, J. Libuda, *Chem. - A Eur. J.* **2020**, *26*, 12445.

[9] H. Ogoshi, Y. Saito, K. Nakamoto, *J. Chem. Phys.* **1972**, *57*, 4194.

[10] J. O. Alben, S. S. Choi, A. D. Adler, W. S. Caughey, *Ann. N. Y. Acad. Sci.* **1973**, *206*, 278.

[11] F. Hoffmann, *Surf. Sci. Rep.* **1983**, *3*, 107.

[12] J. Brede, M. Linares, R. Lensen, A. E. Rowan, M. Funk, M. Bröring, G. Hoffmann, R. Wiesendanger, *J. Vac. Sci. Technol. B Microelectron. Nanom. Struct.* **2009**, *27*, 799.

[13] J. Xiao, S. Ditze, M. Chen, F. Buchner, M. Stark, M. Drost, H. P. Steinrück, J. M.

Gottfried, H. Marbach, *J. Phys. Chem. C* **2012**, *116*, 12275.

[14] D. Wechsler, M. Franke, Q. Tariq, L. Zhang, T. L. Lee, P. K. Thakur, N. Tsud, S. Bercha, K. C. Prince, H. P. Steinrück, O. Lytken, *J. Phys. Chem. C* **2017**, *121*, 5667.

[15] F. Buchner, I. Kellner, W. Hieringer, A. Görling, H. P. Steinrück, H. Marbach, *Phys. Chem. Chem. Phys.* **2010**, *12*, 13082.

[16] M. Chen, X. Feng, L. Zhang, H. Ju, Q. Xu, J. Zhu, J. M. Gottfried, K. Ibrahim, H. Qian, J. Wang, *J. Phys. Chem. C* **2010**, *114*, 9908.

[17] M. E. Bartram, B. E. Koel, *Surf. Sci.* **1989**, *213*, 137.

[18] T. J. Dines, C. H. Rochester, A. M. Ward, *J. Chem. Soc. Faraday Trans.* **1991**, *87*, 1617.

[19] J. Wang, B. E. Koel, *J. Phys. Chem. A* **1998**, *102*, 8573.

[20] R. V. St. Louis, B. Crawford, *J. Chem. Phys.* **1965**, *42*, 857.

[21] F. Hoffmann, *Surf. Sci. Rep.* **1983**, *3*, 107.

[22] M. H. Chang, N. Y. Kim, Y. H. Chang, Y. Lee, U. S. Jeon, H. Kim, Y. H. Kim, S. J. Kahng, *Nanoscale* **2019**, *11*, 8510.

[23] G. Zamborlini, M. Jugovac, A. Cossaro, A. Verdini, L. Floreano, D. Lüftner, P. Puschnig, V. Feyer, C. M. Schneider, *Chem. Commun.* **2018**, *54*, 13423.

[24] S. Stepanow, A. Mugarza, G. Ceballos, P. Moras, J. C. Cezar, C. Carbone, P. Gambardella, *Phys. Rev. B - Condens. Matter Mater. Phys.* **2010**, *82*, 1.

[25] M. S. Liao, S. Scheiner, *J. Chem. Phys.* **2002**, *116*, 3635.

[26] G. Marchand, H. Roy, D. Mendive-Tapia, D. Jacquemin, *Phys. Chem. Chem. Phys.* **2015**, *17*, 5290.

[27] H. Wang, S. M. Butorin, A. T. Young, J. Guo, *J. Phys. Chem. C* **2013**, *117*, 24767.

[28] L. X. Chen, W. J. H. Jäger, G. Jennings, D. J. Gosztota, A. Munkholm, J. P. Hessler, *Science (80-.).* **2001**, *292*, 262.
